# Design of a **syn**demic **bas**ed **in**tervention to facilitate care for men who have sex with men with high risk behaviour: the syn.bas.in randomized controlled trial

**DOI:** 10.1186/s12879-017-2474-x

**Published:** 2017-06-06

**Authors:** Roel C. A. Achterbergh, Jannie J. van der Helm, Wim van den Brink, Henry J. C. de Vries

**Affiliations:** 1 STI outpatient clinic, Department of Infectious Diseases, Public Health Service Amsterdam, Amsterdam, the Netherlands; 2 Department of Psychiatry, Academic Medical Centre, University of Amsterdam, Amsterdam, The Netherlands; 3 Department of Dermatology, Academic Medical Centre, University of Amsterdam, Amsterdam, The Netherlands; 4 Amsterdam Infection & Immunity Institute (AI&II), Academic Medical Centre, University of Amsterdam, Amsterdam, The Netherlands

## Abstract

**Background:**

Men who have sex with men (MSM) constitute a risk group for sexual transmitted infections (STIs), including HIV. Despite counselling interventions, risk behaviour remains high. Syndemic theory holds that psychosocial problems often co-occur, interact and mutually reinforce each other, thereby increasing high risk behaviours and co-occurring diseases. Therefore, if co-occurring psychosocial problems were assessed and treated simultaneously, this might decrease high risk behaviour and disease.

**Method:**

An open label randomized controlled trial will be conducted among 150 MSM with high risk behaviour recruited from the STI clinic of Amsterdam. Inclusion criteria are: HIV negative MSM with two STI and/or PEP treatment in the last 24 months, or HIV positive MSM with one STI in the last 24 months. All participants get questionnaires on the following syndemic domains: ADHD, depression, anxiety disorder, alexithymia and sex- and drug addiction. Participants in the control group receive standard care: STI screenings every three months and motivational interviewing based counselling. Participants in the experimental group receive standard care plus feedback based on the results of the questionnaires. All participants can be referred to co-located mental health or addiction services.

The primary outcome is help seeking behaviour for mental health problems and/or drug use problems. The secondary outcomes are STI incidence and changes in sexual risk behaviour (i.e. condom use, number of anal sex partners, drug use during sex).

**Discussion:**

This study will provide information on syndemic domains among MSM who show high risk behaviour and on the effect of screening and referral on help seeking behaviour and health (behaviour) outcomes.

**Trial registration:**

Trial Registration at clinicaltrail.gov, identifier NCT02859935.

## Background

Men who have Sex with Men (MSM) constitute a risk group for Sexually Transmitted Infections (STI), including HIV. The STI positivity rate – chlamydia, gonorrhea, syphilis and hiv - among MSM attending the STI clinic in Amsterdam is around 18% among HIV-negative MSM and 30% among HIV-positive MSM [1]. Besides this high positivity rate, there is a high recurrence of infections in this group. More than 10% of the men who were diagnosed with an STI had a new infection within one year [1].

For MSM with a high STI risk we started a case holding cohort: the MS2 cohort. We included 107 HIV-negative and 95 HIV-positive MSM from January 2014 till April 2016 [2]. If screened 4 times a year, in 25% of each visit a new STI was found and more then 40% reported using methamphetamine, mephedrone and/or γ-Hydroxybutyric acid (GHB)/ γ-Butyrolactone (GBL) during sex [2]. Despite motivational interviewing and counseling, risk behaviour remains high.

A syndemic is two or more afflictions, interacting synergistically and contributing to an excess burden of disease. The syndemic theory holds that psychosocial problems frequently co-occur, interact and mutually reinforce each other, thereby increasing high risk behaviours and co-occurring diseases [3]. According to the syndemic theory that explains the increased risk on STI in MSM, he following domains can contribute to risk behaviour: drug use, mental health problems, stigma, past or present abuse, and discrimination [4]. Sex related drug use is high among MSM in our MS2 cohort [2], and sex-related drug use is associated with STI [5, 6], HIV [6] and high risk behaviour [7] In addition, MSM report elevated levels of mental health problems, including Attention Deficit Hyperactivity Disorder (ADHD), depressive episodes, obsessive-compulsive disorder and alcohol and drug dependence [8, 9]. If however, co-occurring psychosocial problems were assessed and treated, it might decrease high risk behaviours and co-occurring diseases.

To test this theory we developed the syndemic based intervention (syn.bas.in). The purpose of this open label, randomized controlled intervention at the STI outpatient clinic of Amsterdam is to answer the following question: does syndemic screening and indicated referral to co-located mental health and/or addiction treatment services result in increased help seeking behaviour, and decrease risk behaviour in MSM who are at high risk for STIs and HIV?

## Methods: participants, interventions and outcomes

### Study population

All participants of the MS2 cohort [2] will be invited to join the syn.bas.in study. Other participants will be recruited directly from the STI clinic of the public health center of Amsterdam. Previous studies in the Netherlands have shown that MSM with an STI or a PEP treatment are at high risk of acquiring an HIV infection [10, 11]. Most MSM with HIV have shown high risk behaviour in the past and STI positivity is generally higher then among HIV negative MSM [1]. We expect this intervention to have the most impact on those showing recent high risk behaviour, therefor we choose the following inclusion criteria.

#### Inclusion criteria

The inclusion criteria are MSM, 18 years or older, sufficient command of Dutch or English and

- for HIV positive MSM at least one STI* in the last 12 months.
- for HIV negative MSM at least two STI* or a PEP treatment in the last 24 months.

(* i.e. urethral, pharyngeal or anal gonorrhoea; urethral, pharyngeal or anal chlamydia; lymfogranuloma venereum; syphilis; acute hepatitis B and/or C; and newly diagnosed HIV infection. Genital warts or genital herpes do not count as inclusion criteria).

#### Exclusion criteria

Men will be excluded if they speak insufficient Dutch or English, are not able to complete follow-up or otherwise deemed by clinic staff to be unsuited for participation.

### Study intervention

All participants will receive standard care, including STI screenings every three months and offered a motivational interviewing based counseling session. STI screenings include tests for syphilis, hepatitis B - if not documented vaccinated - hepatitis C, HIV, and anal, urethral and pharyngeal chlamydia and gonorrhea. Asymptomatic men will be screened using a fast track procedure - i.e. no physical examination and self-sampling. Symptomatic men - with suspected STI related signs and/or complaints - will get a full physical examination, including anoscopy. Treatment and contact tracing will be offered to those with a newly diagnosed infection. At the initiative of the participant (e.g. in case of STI related complaints) additional screening will be available.

After explanation of the study by a study healthcare provider and informed consent is obtained, participants will be randomized either to the intervention or the control group. After intake, and at the final visit of the study, all participants fill out a set of questionnaires. Questionnaires will be sent via email and represent various syndemic domains (Table 1).

**Table 1:** Questionnaires on syndemic domains that will be used in the syn.bas.in study for referral of MSM showing high risk behaviour at the STI clinic of Amsterdam

Syndemic domain	Questionnaire	Cut off (range)
Sex addiction	Sexual Compulsivity Scale [15]	≥ 24 (10–40)
Alcohol/drug addiction	AUDIT[17] DUDIT [18]	AUDIT ≥8 (0–12) DUDIT ≥8 (0–40)
Anxiety	Hospital Anxiety Depression Scale [20]	≥8 (0–21)
Depression	Hospital Anxiety Depression Scale [20]	≥8 (0–21)
ADHD	Adult ADHD Self Report Scale [19]	≥4 (0–6)
Alexithymia	TAS-20 [22, 23]	≥52 (20–100)
Partner violence	Stall [13, 24]	/
Childhood sexual abuse	Stall [13, 24]	/
Discrimination	Stall [13, 24]	/

Participants in the intervention arm will be offered the questionnaires a second time before their subsequent three-monthly follow-up visit. During these subsequent visit the outcome of all questionnaires will be discussed to provide the participant in the intervention group a better insight in potential mental and/or addiction problem(s) that possibly contribute to risk behaviour. Via feedback and motivational interviewing the willingness to seek help will be evoked and monitored. All study healthcare providers receive training by mental health care providers to address these issues and are available for consultation in between visits, if necessary. If help is accepted, participants will be referred to a relevant mental health or addiction treatment service. Since co-located care may help to reduce barriers and improve health seeking behavior, intake consultations are available at the STI outpatient clinic of Amsterdam.

Participants in the control group receive standard STI screening and motivational interviewing based counseling session, yet controls are offered the questionnaires only during initial and end visit and do not receive feedback on the results of the questionnaires. Controls are allowed to visit the co-located care. After one year, participants in both groups will be asked to fill out the initial questionnaires again.

### Outcomes

#### Primary outcome

Help seeking behaviour for mental health and/or addiction problems among MSM who are at high risk for STIs and HIV. Since care is co-located, we will be able to use both confirmed help seeking behaviour and self-reported help seeking behaviour (i.e. Have you seen a health professional (family doctor, counsellor or psychologist) to get help for mental problems or problems due to substance use or sex in the last year? yes/no).

#### Secondary outcomes

STI incidence and changes in risky sexual behaviours (i.e. condom use/number of partners/ number of unsafe partners/amount anal sex/recreational drug use/renounce to visit the STI clinic). In addition, we will monitor how care was experienced. If help seeking behaviour took place, information will be collected to know where help was obtained, and how useful the help was on a scale from 1 to 5.

### Study design

**Fig. 1:**
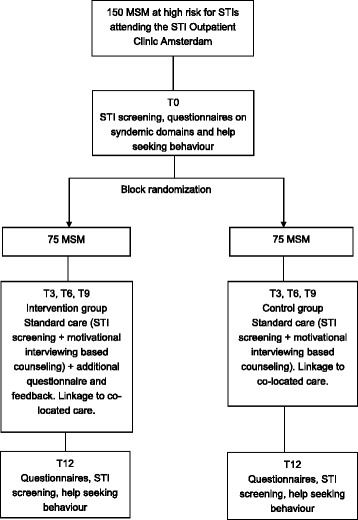
Study design of the syn.bas.in open-label randomized controlled trial with a behavioural intervention to stimulate help seeking behaviour for mental health and addiction problems among MSM at the STI clinic of Amsterdam

This is an open-label randomized controlled trial with a behavioural intervention to stimulate help seeking behaviour for mental health and addiction problems among MSM who show high risk behaviour for STI and HIV at the STI clinic of Amsterdam (Fig. 1). Participants will be assigned by block randomization in groups of 4, 6 or 8, with the help of a computer program.

### Assessments

Previous research has shown associations with HIV positivity and/or risk behavior and several syndemic domains like sexual compulsivity, drug or alcohol use, depression, stress, partner violence, childhood sexual abuse and discrimination [4, 12–14]. Questionnaires on these syndemic domains are added to this intervention. We hypothesized that people with ADHD may engage in risky sexual practices due to problems with impulsivity. Alexithymia might be an important factor associated with depression or anxiety. Therefor alexithymia and ADHD are added on to the list of syndemic domains and the following self-administered questionnaires are used (Table 1).

The Sexual Compulsivity Scale (SCS) is a 10-item self-administered questionnaire that assesses the impact of sexual thoughts on daily functioning and the inability to control sexual thoughts or behaviours [15]. As a cut-off to designate sexual compulsivity (SC), we took a value equal to or above 24 [15].

For alcohol related problems the Alcohol Use Disorders Identification Test (AUDIT) is used. This is a 10–item questionnaire developed for identifying risky or harmful alcohol consumption as well as alcohol-dependence [16, 17].

For drug related problems the Drug Use Disorders Identification Test (DUDIT) is used. This is a 11-item screening instrument developed to identify non-alcohol drug use patterns and various drug-related problems [18].

For ADHD the Adult ADHD self-report scale(ASRS) is used. This is a 6-item screening instrument developed by the World Health Organization to identify adults with ADHD [19].

For anxiety and depression the Hospital Anxiety and Depression Scale (HADS) is used. This is a 14-item screening instrument, 7 items measuring anxiety and 7 items measuring depression [20]. The HADS was found to perform well in assessing anxiety disorders and depression in both somatic, psychiatric, primary care and the general population [21].

For alexithymia the Toronto Alexithymia Scale (TAS) is used. This is a 20-item screening instrument developed to identify persons with alexithymia [22, 23].

Furthermore, we asked for partner violence [13], childhood sexual abuse [13] and discrimination [24] as these variables have been associated with psychosocial health outcomes. (i.e. Did you experience any form of violence (symbolic (e.g., verbally, someone threatened you, stalked you) physical (e.g., someone hit you, kicked you) or sexual(e.g., someone forced you to have sex)) in the past 5 years with a primary partner?; Did you ever experience being ‘forced or frightened’ by someone into doing something sexual with a partner who was more then 10 years older at the moment when you were 16 years or younger?)

Tests used for STI screening in routine practise at the STI outpatient clinic of Amsterdam will be used in the study. The APTIMA ® Combo 2 assay is a Nucleid acid amplification test (NAAT) used to detect RNA of both *Chlamydia trachomatis* and *Neisseria gonorrhoeae* in the urethra, rectum and pharynx. The INNO-LIA Syphilis Score, Fujirebio/Innogentetics is used for syphilis serology. The Liaison® XL murex is used to detect antibodies for hepatitis B and hepatitis C. Both an Alere Determine HIV 1/2 rapid test and a 4th generation combo test (INNO-LIA™ HIV I/II Score, Fujirebio) are used for HIV screening.

Clinical data and biological materials will be stored according to the STI clinic procedures. Electronic patient files are accessible to all medical personnel of the STI clinic with access and codes to this system. All data gained from questionnaires will be kept under a unique project ID, which will be given at each participant at the beginning of the study based on the number of previous participants. A master list linking study ID’s to electronic patient file identifiers will be controlled by the study coordinator. The list will be kept in a locked filing cabinet with access restricted to the study coordinator.

### Sample size calculation

In the Amsterdam health monitor, 7% of the MSM respondents reported to have serious psychosocial problems [25]. Because of the lack of data, we hypothesize that 7% of the participants will already be in care for psychosocial problems. With our intervention we intend to increase help seeking behaviour from 7% to at least 25%. As 89% of the MS2 cohort used at least two different drugs [2], we assume that at least 40% will score above the DUDIT or AUDIT cut-off. Combined with the other questionnaires, including the ADHD, sexual compulsivity, alexithymia, anxiety and depression questionnaires, we expect that about 50% of the study population will score above the cut off of at least one syndemic domain questionnaires. Previous studies have shown that MSM at the STI clinic are willing to participate in STI clinic initiatives. Using the opting-out strategy for HIV-testing, refusals to test for HIV dropped from 38% to 2% within one year [26]. In the syn.bas.in study we will actively stimulate help seeking behaviour. Therefore we believe that an improvement in help seeking behaviour from 7% to at least 25% is a realistic estimate. To detect an increase of help seeking behaviour from 7% to 25% and a statistical power of at least 80%, at least 64 participants per group are needed. Taking into account 10% loss to follow up, at least 141 participants are needed. Our aim is therefore to recruit 150 participants.

### Statistical analysis

Descriptive statistics will be used to describe baseline characteristics, questionnaire scores, condom use, number of partners, number of unprotected anal sex partners, recreational drug use (during and outside sexual activities) and lost to follow up rate. The distribution of the characteristics of the two intervention groups will be compared using Chi-squared tests for categorical data and student t-test or rank sum tests for continuous data depending on the distribution of the characteristics. To assess to effect of the intervention (control group vs. experimental group) on the primary outcome variable (help seeking behaviour), a univariable logistic regression analysis will be performed. Similar analyses will be performed with the secondary outcome variables. Changes over time will be described for the following parameters: drug use, risk behaviour and incident STIs. We will use appropriate univariable and multivariable statistical methods (poisson regression), corrected for repeated measurements within individuals to investigate changes over time and associated determinants. These analyses will be conducted comparing the two intervention groups.

## Discussion

The syn.bas.in study will provide information on the psycho-social status of high risk MSM in an STI clinic setting in Amsterdam, The Netherlands. Several syndemic domains will be explored in a multidimensional fashion and provide a broader view on the potential issues that contribute to STI risk behaviour. More importantly, it will assess the effect of a psycho-social self assessment procedure with subsequent feedback and active referral among MSM with one or more high syndemic domain problem scores. This may lead to further care-optimization for MSM, especially those who show high risk behaviour for STI and HIV. If this study is successful it may also be used for further research in other settings frequented by high risk MSM like HIV treatment centers. Moreover, the data generated can be used to further explore the value of the syndemic theory and eventually reduce STI and HIV infections.

### Strengths

A strength of this study is that we will be able to test for both STI and risk behaviour, whereas many other intervention studies do not have laboratory confirmed STI outcomes [27]. Also, by using computer-assisted self-interviewing the reliability and validity of the generated data on risk behaviour will be as unbiased as possible. Another strength is that the study team is well trained in motivational interviewing which is also used in addiction care. This might help during the referral process and follow-up period. Finally, all the care provided is from trained and established organizations. Bringing STI, drug use and mental health care together is innovative and the co-located care might help participants to overcome barriers to seek help for problems which can be difficult to address. Incorporating these 3 echelons (STI, drug use and mental health care) within one program might be an answer to meet the emerging rise of chemsex and the associated physical, mental and social problems [28].

### Limitations

The trial may have some limitations. First, this is an open label trial and thus the study team can be influenced in its evaluation of the outcomes resulting in information bias and an overestimation of the effect of the intervention. We cannot control for this bias. However, most questionnaires used to assess the outcome measures are validated, will be self administered and therefore cannot be influenced by the study team. Second, co-located mental health and addiction care will not only be available to participants in the intervention group, but also for participants in the control group as it seems unethical not giving them this opportunity. This may result in weakening of the contrast and thus an underestimation of the effect of the intervention. However, participants in the control group will not be stimulated by the study team. Third, the study is only performed in one center. In the future, it would strengthen our research and our findings if this study is performed in more centers and countries. Also it would strengthen the syndemic theory if we would use STI as primary outcome. As treatment for psychosocial problems can take a long time and therefore the effect on STI incidence as well, we decided to use help seeking behaviour as our primary outcome and STI as secondary outcome.

## Abbreviations

ADHD: Attention Deficit Hyperactivity Disorder
ASRS: Adult ADHD Self-Report Scale
AUDIT: Alcohol Use Disorder Identification Test
DUDIT: Drug Use Disorder Identification Test
GBL: γ-Butyrolactone
GHB: γ-Hydroxybutyric acid
HADS: Hospital Anxiety and Depression Scale
HIV: Human Immunodeficiency Virus
MS2: A cohort of men who have sex with men showing high risk behavior at the sexually transmitted infections clinic in Amsterdam
MSM: Men who have Sex with Men
NAAT: Nucleic Acid Amplification Test
PEP: Post-Exposure Prophylaxis
SC: Sexual Compulsivity
SCS: Sexual Compulsivity Scale
STI: Sexually Transmitted Infection
TAS: Toronto Alexithymia Scale
